# Connecting breast cancer survivors for exercise: protocol for a two-arm randomized controlled trial

**DOI:** 10.1186/s13102-021-00341-w

**Published:** 2021-10-14

**Authors:** Jenna Smith-Turchyn, Michelle E. McCowan, Erin O’Loughlin, Angela J. Fong, Meghan H. McDonough, Daniel Santa Mina, Kelly P. Arbour-Nicitopoulos, Linda Trinh, Jennifer M. Jones, Jackie L. Bender, S. Nicole Culos-Reed, Jennifer R. Tomasone, Madison F. Vani, Catherine M. Sabiston

**Affiliations:** 1 School of Rehabilitation Science, McMaster University, 1400 Main Street West, Hamilton, ON L8S 1C7 Canada; 2 Faculty of Kinesiology and Physical Education, University of Toronto, Toronto, Canada; 3 Department of Kinesiology and Health, Rutgers University, New Brunswick, USA; 4 Faculty of Kinesiology, University of Calgary, Calgary, Canada; 5 Princess Margaret Cancer Centre, University Health Network, Toronto, Canada; 6 School of Kinesiology and Health Studies, Queen’s University, Kingston, Canada

**Keywords:** Exercise, Physical activity, Peer-based interventions, Social support, Breast cancer

## Abstract

**Background:**

Peer-based exercise interventions that cultivate new opportunities for support with a fellow cancer survivor may result in increased exercise volume. It is not clear whether adding qualified exercise professional (QEP) support to peer-based interventions improves health outcomes. Therefore, the purpose of this study is to determine whether breast cancer survivor (BCS) dyads who receive 10 weekly sessions of virtually delivered QEP support have improved outcomes compared to BCS dyads who do not receive QEP support.

**Methods:**

*Participants* Adult BCS with medical clearance for exercise, who have an internet-connected device, and currently engage in < 150 min of moderate-intensity exercise per week. *Intervention* BCS will be matched using evidence-based criteria. The intervention group will receive dyadic exercise information sessions and a program tailored by a QEP for 10 weeks (intervention period) and have access to the QEP for an additional 4 weeks (tapering period). The control will not receive any QEP support. *Outcomes* The primary outcome is post-intervention self-reported exercise volume. Secondary outcomes include device-assessed exercise volume (i.e., Fitbit), social support, and health-related quality of life. *Randomization* 108 participants, matched in dyads, will be randomized 1:1 to the *MatchQEP* or *Match* groups using a web-based scheme. *Statistical analysis* Outcomes will be measured at baseline, post-intervention, post-tapering, and at 12 weeks post-intervention follow-up.

**Discussion:**

The findings from this RCT will determine if matched BCS dyads who receive 10 weeks of virtually delivered QEP support have higher levels of self-report and device-measured exercise, social support, and health related quality of life compared to matched dyads without QEP-delivered exercise guidance. To our knowledge this will be the first study to assess the combined effect of peer- and QEP support on exercise volume. Project findings will inform and optimize intervention methods aimed to increase exercise among BCS through accessible exercise supports.

*Trial Registration*: The study is registered on ClinicalTrials.gov (study identifier: NCT04771975, protocol Version Number: 2, date: July 22, 2021).

**Supplementary Information:**

The online version contains supplementary material available at 10.1186/s13102-021-00341-w.

## Background

One in eight women in Canada will be diagnosed with breast cancer in their lifetime [1]. Despite increasing survival rates [1] and attention to managing the sequelae of breast cancer, survivors face numerous physical and mental health threats during and after primary treatment that impact their daily functioning and health-related quality of life (HRQOL) [2–5]. Exercise is well-established as a safe and feasible means of improving health and wellbeing through all phases of breast cancer survivorship, including for individuals living with metastatic disease [5–13]. There is long-standing evidence from randomized controlled trials (RCTs) involving breast cancer survivors (BCS) that exercise improves physical, mental, and social health indicators linked specifically to HRQOL; and that exercise is associated with reduced risk of cancer recurrence, cancer-specific mortality, and all-cause mortality [5–15]. Cancer exercise guidelines recommend that BCS engage in a minimum of 90–150 min of at least moderate intensity exercise per week and resistance training at least two times per week [16–18]; however, the majority of BCS do not meet these guidelines [19–22].

In addition to the common environmental and health system exercise barriers that people without cancer face, BCS report that the physical effects of breast cancer treatment pose an additional barrier to exercise [20, 23, 24]. Lack of social support and a lack of access to appropriate exercise programming also prevent engagement in exercise among BCS [20, 23, 25, 26]. For survivors in ‘hard to reach’ demographic groups (e.g., young adults, those living in rural communities, and/or in an area with low socioeconomic status), finding age-appropriate, affordable, and accessible exercise support is particularly challenging [25].

Social support is positively associated with exercise among BCS [27–29], but few studies have included a systematic examination of the influence of different sources and forms of support within exercise interventions (e.g., professional support vs. spousal support/caregiver support vs. support from other survivors) [30–32]. The amount and quality of exercise-related support received has focused on either tangible assistance (e.g., people giving you materials or products that help you to exercise), emotional support (e.g., people providing empathy or care when you discuss the difficulties of exercise), informational support (e.g., people providing information on the benefits of exercise), or esteem support (e.g., people providing encouragement that helps you to exercise) [30]. While these different types of support can come from a variety of sources within a breast cancer survivor’s social network (e.g., family, friends, colleagues, trusted health professionals, and support group members), peer-to-peer support external to an existing social network has not been studied in depth. Drawing from existing evidence, peer-based interventions that intentionally cultivate new opportunities for support (i.e., receiving support from a peer that is outside of, or in addition to, one’s existing support network [33]) can result in positive changes to exercise behavior that can match the effectiveness of professionally delivered supports [34, 35]. Furthermore, peer support interventions that can be delivered remotely have been successful in increasing moderate to vigorous physical activity (MVPA) among BCS [36, 37]. Unfortunately, BCS still report unmet needs related to their ability to find an exercise support peer, especially one who shares the lived experience of cancer [38]. BCS also report challenges with accessing support for exercise from qualified health professionals [24, 25].

Online exercise peer matching systems have been developed specifically for women with cancer in an effort to mitigate social support-related barriers to exercise (see ActiveMatch [activematch.ca] and 2Unstoppable [2unstoppable.org]). The peer-to-peer relationship offers unique benefits and is likely to involve different pathways to exercise initiation and maintenance in comparison to professionally delivered exercise support [35]. Nonetheless, even if two survivors are well-matched as exercise peers, this form of peer-to-peer support does not directly address the barrier of a lack of access to exercise guidance from qualified health professionals [39]. Undoubtedly, the relationship between two untrained peers (two survivors) is distinct from the relationship that exists between a survivor and a qualified exercise professional (QEP). As such, the addition of QEP support has the potential to address survivors’ desire for tailored exercise guidance and synergistically enhance the benefits of receiving social support from a well-matched exercise peer. Furthermore, exercise guidelines for cancer survivors specify the importance of supervised exercise programming [16–18], and suggest that QEP support may be most beneficial for BCS who are the least active and who have the most to gain from exercise support [40].

**Fig. 1:**
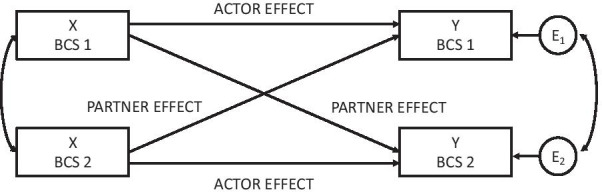
Actor-Partner Interdependence Model framework for indistinguishable dyads testing the effects of breast cancer survivor peers on moderate-to-vigorous physical activity (X = Baseline, Y = Post-10 week intervention as primary endpoint)

In the current study, the primary aim is to examine the independent effects of matched peers on self-reported exercise volume among BCS in a RCT with one group of dyads assigned to receive QEP support and the other group of BCS dyads not receiving QEP support. Secondary aims include exploring the effects of matched peers on device-measured moderate-vigorous physical activity (e.g., Fitbit), perceived social support, and HRQOL. Actor-Partner Interdependence models (APIM) (Fig. 1) will be used as a framework to study the peer (dyadic/partner) effects and the effects of the intervention (QEP support) over time [41–43]. Also, a strategic assessment of the cost-effectiveness of offering virtually delivered QEP sessions to matched BCS dyads will help to inform and optimize methods of increasing exercise among BCS through accessible exercise supports.

### Study purpose

The purpose of this study is to examine interpersonal and individual effects of partnering BCS with a peer and a QEP on self-reported exercise volume (MVPA; primary outcome), and device-measured exercise volume (MVPA; Fitbit), social support, and HRQOL (secondary outcomes). Cost-effectiveness and intervention adherence will also be explored as tertiary outcomes. The effects of a peer and QEP-support intervention group, labelled *MatchQEP*, will be compared to a control group of BCS who are matched with a peer, but not a QEP, labeled *Match*.

There are three main research questions and hypotheses (also presented in Table 1):

1. Are there any differences between the *MatchQEP* group and the control group on self-reported MVPA after the intervention (10 weeks post baseline)? Based on the consistent evidence for positive effects of exercise specialists on MVPA, we expect the peers in the *MatchQEP* group to report greater increases in MVPA than the peers in the control group at post-intervention. We expect these differences to be maintained at post-tapering and follow-up data collections.
2. Does exercise peer 1’s exercise volume (minutes of MVPA) at baseline predict exericse peer 2’s exercise volume after the intervention (and vice versa)? A positive peer effect between baseline and post-intervention within the *MatchQEP* group is expected as the QEP is expected to work with the dyads in ways to support each other’s MVPA. In contrast, non-significant or small peer effects are expected to be observed within the control group who receive no QEP support.
3. To what extent does MVPA at baseline predict MVPA after the intervention? The QEP is expected to improve MVPA and the effects are likely to be strongest for the most inactive women. Therefore, the stability of individual differences in MVPA may be lower in the group that has QEP support compared to the control group.

**Table 1:** Research questions and hypotheses for the main study effects exploring the impact of partner (i.e., peer) effects and qualified exercise professional (QEP) support on physical activity among breast cancer survivors

Type of effects	Explanation	Hypotheses
Interpersonal effects		
Partner effect _(P1–P2)_	Influence of the peer 1 (P1)'s baseline MVPA on the peer 2 (P2)’s post-intervention MVPA	Positive effect in intervention group, no effect in control group
partner effect _(P2–P1)_	Influence of the P2’s baseline MVPA on the P1’s post-intervention MVPA	Positive effect in intervention group, no effect in control group
Individual effects		
Actor effect _(P1)_	Stability of the P1’s MVPA from baseline to post-intervention	Intervention group < control group
Actor effect _(P2)_	Stability of the P2's MVPA from baseline to post-intervention	Intervention group < control group
Treatment effect _(P1)_	Difference between the P1’s mean post-intervention MVPA of the intervention group and of the control group	Intervention group > control group
Treatment effect _(P2)_	Difference between the P2’s mean post-intervention MVPA of the intervention group and of the control group	Intervention group > control group
Intervention effects		
Adherence	Adherence is established if BCS in the *MatchQEP* group attend at least 7 out of 10 assigned sessions with the QEP Adherence to peer support in both *MatchQEP* and *Match* groups will be established based on the number of times the peers connect (expecting at least once per week for 10 weeks)	Adherence will be established
Cost-effectiveness	Direct costs will be compared between the *MatchQEP* and *Match* groups and between the *MatchQEP* group to traditional QEP services in Canada	*MatchQEP* will be cost-effective

All research questions also align with the secondary outcomes, such that device (e.g., Fitbit) measured MVPA, social support, and HRQOL will be higher among the *MatchQEP* group.

Intervention adherence and cost-effectiveness will also be explored. We expect BCS to adhere to the *MatchQEP* group by attending at least 70% of the sessions [44, 45] and BCS to adhere to the *Match* group by connecting with their partners at least once per week for 10 weeks. We expect the *MatchQEP* group to be cost-effective (e.g., have lower direct costs compared to traditional in person and clinical QEP services in Canada and a favorable incremental cost-effectiveness ratio compared to the *Match* group). The *MatchQEP* group is also expected to report less health care use (e.g., health care facility visits, doctor visits, procedures received, support services used, and loss of work) compared to the *Match* group.

## Methods

### Study design and participants

**Fig. 2:**
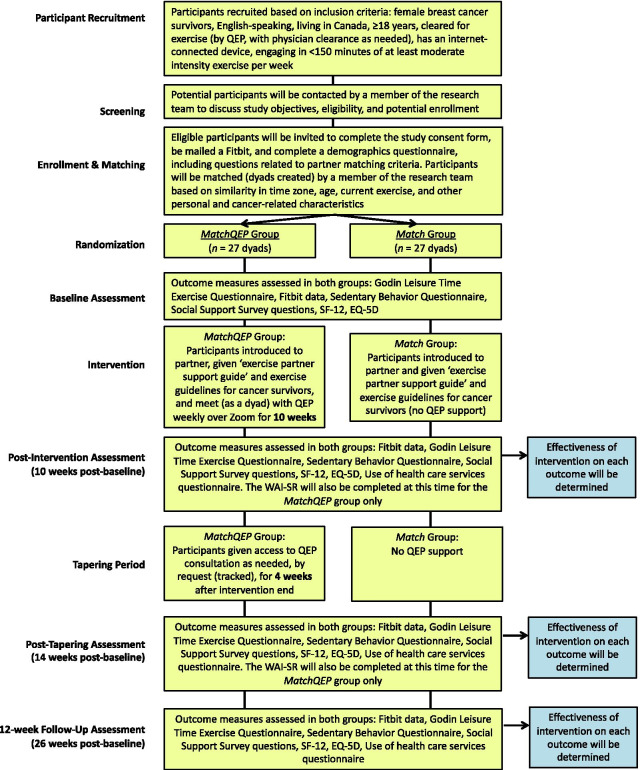
Study flow

This is a two-arm RCT with validated outcome assessment measures. The protocol adheres to the CONSORT guidelines (Fig. 2) [46] and SPIRIT recommendations for reporting of clinical trial protocols [47]. Eligible participants are: (1) English-speaking female BCS; (2) diagnosed with primary stage 0–IV breast cancer, at any stage of treatment; (3) living in Canada; (4) aged 18 years or older; (5) medically cleared for exercise; (6) connected to the internet using any device (e.g., computer, tablet or smartphone; preferably with webcam); and (7) currently engaging in less than 150 min of MVPA per week [16–18]. Participants will be excluded from the study if they report any contraindications to exercise, or had recent or have planned surgery of any kind (including reconstructive surgery).

### Recruitment

BCS will be recruited using digital recruitment materials. Study investigators will send a recruitment email and social media posts to select community-based cancer programs, services, and organizations across Canada for administrators at these organizations to share with their community members. All recruitment materials will include instructions for interested individuals to contact the study team directly. Eligibility will be confirmed by email or phone call by a member of the study team and all eligible participants will provide informed consent prior to enrollment. The University of Toronto’s Human Research Ethics Unit has approved this study (protocol #00038665).

### Sample size

Sample size calculation was carried out based on APIM power analysis for indistinguishable dyads [48]. Specifically, the alpha was set to 0.05 and the sample size estimate is the smallest number of dyads required to detect the effect when power is at least 0.8. Based on standardized regression coefficient effect size estimates (beta; *ß*) drawn from a pilot study of 46 women matched for exercise (23 dyads) assessed over 21 days (i.e., reflecting the *Match* group in the current study), the actor effect size was *ß* = 0.33, the partner effect size was *ß* = 0.28, and the correlation between the two women’s self-report scores on minutes of exercise at baseline was *r* = 0.46. The correlation of the errors was set at 0.30. At minimum, 32 total dyads are needed to have adequate power to detect an actor effect and 43 total dyads are needed to have adequate power to detect a partner effect for a required sample size of *N* = 86. Attrition may be 30% [49], and 11 additional dyads will be recruited for a total sample size of 108 women (54 dyads total across both groups).

### Treatment allocation and randomization

Dyads will be randomized (1:1) to the intervention (*MatchQEP*) or control (*Match*) group prior to baseline assessment. Treatment allocation will be centrally randomized using a web-based scheme (https://www.randomizer.org/). The randomization will be completed by a PhD student external to the research team and group allocation will be assigned in the order in which participants have completed an initial background and demographics questionnaire and have been matched with a peer for the study. Although the research coordinator(s), QEPs, and participants cannot be blinded to treatment allocation, the primary investigators and statistician completing data analysis will be blinded.

### Intervention

Matching of participants into dyads will be conducted by a research coordinator based on evidence-informed criteria developed in our formative study [50]. For participants to be matched, they must: (1) live in the same time zone, (2) be in the same age range (i.e., within ± 10 years of age), and (3) self-report similar current exercise volume (i.e., within 30 min per week). Beyond these core matching criteria, we will create discrepancy scores based on other personal and cancer-related characteristics, such as cancer stage at diagnosis, treatment status, time since treatment, participants’ family situation (e.g., having a romantic partner and/or children), and exercise preferences (e.g., preferred time of day for exercise) [25, 51] and account for discrepancies in these characteristics in analyses. All participants will receive an ‘Exercise Peer Support Guide’ that provides tips and strategies for supporting their exercise peer [52, 53] and a one-page infographic highlighting current exercise guidelines for cancer survivors [16–18]. All participants will be given a Fitbit wearable activity tracking device, which will be used for device-measured MVPA. Matched exercise peers will be introduced to one another by a research assistant via Zoom (video conferencing platform) prior to the start of the study period. Refer to Fig. 2 for a study flow chart.

#### Intervention condition

Participants in the *MatchQEP* group will receive exercise information and program sessions tailored by a QEP specifically for each BCS in the dyad. A single QEP, with advanced training on exercise for individuals with cancer, will provide all QEP sessions for each dyad. The study QEP also has research experience leading longitudinal and experimental trials while also holding a PhD in behavioral medicine. *MatchQEP* dyads will meet with the QEP via Zoom once per week for 10 weeks. Each weekly session will last up to 60 min and will include a review of the exercise program, achievement of goals/action plans set, adverse effects/events, and discussion on overcoming barriers to completing the prescribed exercise program. The actual length of the session, in minutes, will be noted. Additionally, the QEP will follow a standardized guide to deliver specific sessions each week based on behavior change counselling support strategies [54, 55] (see “Appendix”). Based on current exercise recommendations for cancer survivors [16–18], the overall goal of the QEP-prescribed exercise intervention is for each member of the dyad to complete a minimum of 150 min of MVPA per week, which may include both aerobic and resistance forms. Consistent with home-based exercise strategies, the QEP will tailor the program based on personal circumstances, including cancer-related characteristics, side effects, current fitness level, and available space and equipment [16]. *MatchQEP* group participants will have the opportunity to reach out to the QEP individually if they need to ask specific and personal questions during the intervention period. For 4 weeks following the completion of the 10-week intervention, the QEP will be available for consultation (i.e., a post-intervention tapering period) as needed by the *MatchQEP* group participants. This tapering period is important for understanding strategies to successfully taper BCS from exercise trials. All interactions between the QEP and the dyads from intervention initiation until the end of the tapering period will be recorded.

#### Control condition

BCS in the *Match* (control) group will independently communicate and support each other around exercise for 10 weeks. They will not have any contact with a QEP during this time and will structure their own communication with their matched peer. After the 12 weeks post-intervention follow-up assessment (26 weeks post-baseline), BCS in this group will be offered a single consultation session with a QEP to discuss exercise-related questions.

### Data collection and measures

Following informed consent, all BCS will complete a demographics questionnaire, including questions related to peer matching criteria, treatment, and exercise history. To determine the effectiveness of the intervention, BCS will complete exercise volume, social support, and HRQOL questionnaires online at four time points: baseline, post 10-week intervention, post 4-week tapering (i.e., 14 weeks post-baseline), and at 12 weeks post-intervention follow-up (i.e., 26 weeks post-baseline). BCS will be asked to wear the Fitbit to collect device-measured MVPA for one week at all four timepoints. Self-reported sedentary behavior will be assessed as a potential covariate at all time points. The QEP alliance (i.e., connection to the QEP support provider) will be assessed as a covariate at post-intervention and post-tapering time points. Adherence to the intervention will be calculated at the end of the intervention period (10 weeks) and cost-effectiveness will be assessed at post-intervention (10 weeks), post-tapering (14 weeks), and at the 12-week post-intervention follow-up (26 weeks after baseline assessment).

### Primary outcome

Exercise volume at the post 10-week intervention time point, will be measured using a valid and reliable self-report survey (modified Godin Leisure Time Exercise Questionnaire) [56–58]. The modified Godin Leisure Time Exercise Questionnaire is a self-report measure which asks respondents to give weekly frequencies and durations of strenuous, moderate, and mild aerobic activities. Resistance training volume will also be self-reported using the same format as the aerobic activities (e.g., frequency and time metrics). Responses on moderate and strenuous activities are summed to determine total MVPA volume. This measure will also be assessed at the post-tapering (i.e., 14-weeks) and 12 weeks post-intervention follow-up (i.e., 26 weeks post-baseline) time points.

### Secondary outcomes

Device-measured MVPA, social support, and HRQOL are secondary outcomes.

#### Device-measured MVPA

Exercise volume will also be assessed using a tracking device (Fitbit Inspire® 2 accelerometer). Adherence to Fitbit’s use in cancer survivors is high [59, 60] and Fitbit exercise data has demonstrated high correlation to Actigraph measures in this population [59]. Fitbit devices will be mailed to BCS at study inception and will be required to be worn for 7 consecutive days during the four primary data collections to determine their average daily and weekly 

s of MVPA and step count. BCS are not required to wear the device outside of the data collection timeframes, but can wear them if they choose. Total wear time and corresponding data will be collected by a research assistant via the online Fitbit database in each participants unique, deidentified study Fitbit account and will be used to characterize the use of Fitbit devices over the entire study duration. Participants will keep the devices post-intervention.

#### Social support

A breast cancer-specific version of the Social Support Survey (SSS) [31, 61, 62] will be used to assess seven dimensions of social support: listening support, task challenge, emotional support, esteem support, reality confirmation, tangible assistance, and understanding breast cancer support [31, 61, 62]. Within each dimension, respondents will be asked to score two questions on a five-point Likert scale (amount (total number) and satisfaction with each type of support rated from very dissatisfied to very satisfied). The SSS has demonstrated adequate construct validity in sport settings [57] and has been validated in a sample of BCS [63]. Participants will also rate the amount (total number) and perceived quality (from very dissatisfied to very satisfied) of support they receive in the following exercise-related support categories: listening support, task challenge, emotional support, esteem support, reality confirmation, tangible assistance, and informational support. At the post-intervention, post-tapering, and follow-up assessments, participants will rate, on a seven-point scale ranging from 1 (none at all) to 7 (a lot), the amount of each type of exercise-related support that they received from their study exercise partner since the last assessment time. For the purpose of this study, a total social support latent variable will be estimated to account for general and exercise-specific social support. See Additional file 1.

#### HRQOL

The Short-Form-12 (SF-12) [64, 65] and the EQ-5D-3L [66] will be used to assess HRQOL. The SF-12 is a self-administered questionnaire consisting of 12 items addressing eight domains of health (physical functioning, role-physical, bodily pain, general health, vitality, social functioning, role-emotional, and mental health). The SF-12 has demonstrated validity in adult populations when compared to the SF-36, with high correlations for both the physical (*r* = 0.94–0.96) and mental (*r* = 0.94–0.97) domains, showing that it is a practical alternative to the longer SF-36 [65]. The EQ-5D-3L [66] is a two-part measure. The first part using a three-level scale to assess five dimensions of health (mobility, self-care, usual activities, pain/discomfort, and anxiety/depression). The second part assesses responder’s perception of their health on a visual analogue scale from 0 (worst imaginable health) to 10 (best imaginable health). This measure is reliable and valid for individuals with cancer [67]. For the purpose of this study, a total latent variable for HRQOL will be tested and used in the main analysis exploring HRQOL effects as a secondary outcome of the study.

### Tertiary outcomes

#### Intervention adherence

The QEP will track *MatchQEP* group adherence by completing a weekly session log. The QEP will record attendance of each BCS at the virtual QEP session as well as whether each individual completed the goals set from the previous session. *MatchQEP* group adherence is defined as the number of sessions attended divided by the total number of sessions. Adherence in the *Match* group will be determined by the number of times the peers connect during the 10-week intervention period.

#### Cost-effectiveness

The costs of the *MatchQEP* intervention will be calculated and compared to traditional face-to-face costs of QEP services in Canada and to the *Match* group alone. Refer to Table 2 for a description of costs to be compared between *MatchQEP* group and traditional QEP services. The use of health care resources will also be compared between the two groups at the post-intervention, post-tapering, and follow-up time points using a piloted questionnaire [68] that assesses health care facility visits, doctor visits, procedures received, support services used, and loss of work (see Additional file 2).

**Table 2:** Cost of *MatchQEP* group versus traditional QEP services

*MatchQEP group*		
Program costs^#^	Professional time/labor	# of contact hours × unit cost of Qualified Exercise Professional (QEP) over the 10-week intervention period and 4-week tapering period*
	Equipment	Any device a survivor may purchase (pay out of pocket) for use at home based on the QEP’s recommendation (such as TheraBand or free weights) Computer for QEP
*Traditional QEP services*		
Session costs^#^	Total cost of QEP session	Includes venue use/cost of membership, equipment at venue, preparation time for the QEP
	Labor	# of QEP sessions attended during a comparable 10-week period × cost of each session
Travel	Mileage estimates	Based on distance (km) from an individual’s home to the session venue and the cost of gas (/km)
	Public transportation costs	
	Parking costs	
Home-based equipment		Any form of device that a survivor may purchase for use at home based on the QEP’s recommendations (for example, TheraBand or free weights)
Consumable materials	Photocopies of home exercises	
	Printed letters/folders given to survivors	

^*^For each 1-h contact cost, 1.5 h of labor will be allocated to account for the preparation time required for each session

^#^The cost of training staff will be excluded as staff will already be qualified for their role

### Descriptive data and covariates

Age in years, stage of cancer, time since treatment, time since diagnosis, current medications, and exercise preferences will be self-reported at baseline in an online demographic questionnaire. The Sedentary Behavior Questionnaire [69] will be used to assess the amount of time (in hours) per week spent engaged in sedentary pursuits (e.g., watching television, sitting and reading). The scale has demonstrated reliability and validity in adult populations [69]. Sedentary behavior will be a covariate [70]. Exercise peer match quality (assessed on a 5-point scale from 1: very poor match to 5: very good match), amount of time spent with peer, method of connecting with peer, and perceived similarities with peer (assessed on a 5-point scale from not at all similar to extremely similar) will be collected post-intervention and used as covariates in analyses (see Additional file 3). Therapeutic alliance between participants and the QEP will be assessed by each participant in the *MatchQEP* group at the post intervention (10 week) and post tapering (14 week) time points using the modified Work Alliance Inventory—Short Form Revised (WAI-SR) [71]. The WAI-SR is a twelve-item scale evaluating three domains of therapeutic alliance: (1) agreement between patient and therapist on the goals of treatment, (2) agreement between patient and therapist about the tasks to achieve these goals, and (3) the quality of the bond between the patient and therapist (QEP) [71]. Each item is assessed on a 7-point Likert scale from 0 (never) to 7 (always), with higher scores representing higher satisfaction with the therapist (QEP)-patient relationship [65]. The WAI-SR is a reliable and valid outcome for assessing therapeutic alliance (internal consistency: α = 0.91, test–retest reliability: *r* = 0.93; and construct validity: *r* = 0.74) [71].

#### Safety/adverse events

While exercise is known to be safe for BCS when performed correctly [14, 15], minor injuries have the potential to occur in any exercise intervention. To enhance participant safety in this study, all participants will be cleared to exercise prior to enrollment using the PAR-Q+/ePARmed-X+, [72] with physician clearance as needed. For the women in the *MatchQEP* group, tailored exercise recommendations will be provided during the exercise program delivery. Any injuries or side effects that occur in the *MatchQEP* group will be tracked by the QEP on an ‘events’ log with all adverse events classified on severity with specific types/class using the “Common Terminology Criteria for Adverse Events”. Adverse events will be reported to the Principal Investigator immediately for follow-up with BCS and connection to physician for further consultation.

### Statistical analysis

All analyses will be conducted using R [73] and MPlus 7.4 [74]. Significance will be established at *p* < 0.05 for all coefficients. Descriptive statistics (means, frequencies, zero-order correlations) will be computed for all measures over time. The main analysis will be guided by APIM [41] to explore interpersonal and individual effects of the intervention (QEP support) for the primary and secondary outcomes over time.

In the APIM (see Fig. 1), effects of both dyad members are assessed simultaneously by estimating interpersonal peer effects (labelled partner effects in APIM) and individual effects (labelled actor effects in APIM). Three types of effects can be used to explore the research questions examining MVPA among BCS. First, the peer (i.e., partner) effects indicate the average influence of each BCS’s MVPA in the dyad. Second, the actor effects assess the average stability of MVPA across the intervention for each dyad member. Specifically, actor effects provide an indication to the effectiveness of the QEP support intervention since MVPA is assumed to be altered by QEP support more strongly when compared to the control group. Third, the average intervention effects will be tested and compared to post-intervention MVPA. The effectiveness of matching BCS dyads on device-measured MVPA, social support, and HRQOL will also be explored in separate models testing actor and partner effects on these secondary outcomes.

Multiple-group structural equation modeling (SEM) will be used to estimate and test an APIM with two groups (*MatchQEP* vs. *Match*). To address research question 1, the mean post-intervention levels of MVPA (controlling for actor and partner effects) of the two groups will be compared. For research question 2, the average partner (i.e., peer) effects of MVPA from each BCS in the dyad will be estimated within each group [43]. The effects will be tested by comparing constrained and unconstrained models on chi-square goodness-of-fit values. For research question 3, the average actor effects for MVPA among BCS will be estimated for the *MatchQEP* and *Match* groups and will be tested against each other. These analyses will be repeated for device-measured MVPA, social support, and quality of life (secondary outcomes). Measured covariates (e.g., cancer and personal characteristics, partner characteristics and quality of match, therapeutic alliance) will be included in the models as necessary based on their association with the outcomes.

To assess the intervention effects (tertiary outcomes)*,* adherence will be examined based on the number of sessions attended with the QEP in the *MatchQEP* group and on the frequency the peers connected over the duration of the study for both *MatchQEP* and *Match* participants. Cost-effectiveness will be determined by comparing the direct intervention costs between study groups and between the *MatchQEP* group and traditional, in-person QEP services. Additionally, cost-effectiveness of the intervention group, compared to control group, will be calculated based on the intervention costs per participant with respect to changes in the assessed health outcomes. Incremental cost-effectiveness ratios will determine the cost of one unit of change for the investigated outcomes. Mean costs of each category of health resource use (health care facility visits, doctor visits, procedures received, support services used) and loss of work will be calculated for each group and compared for significant differences using *t* tests. All costs will be determined based on current 2021 Ontario Health Care standards in Canadian dollars.

## Discussion

The benefits of exercise for BCS span physical, psychological, and social health [5–11]. Regular exercise also has the potential to decrease mortality in BCS [12–14]. Due to the low rates of health enhancing MVPA among BCS [19–21], efforts are needed to help BCS begin and sustain optimal activity levels. Currently, BCS report a lack of support for exercise and lack of information from a QEP as barriers to exercise participation [20, 23]. Tailored exercise information from a QEP and social support, specifically from fellow survivors, are common requests from BCS in order to facilitate engagement in exercise at various points in the survivorship trajectory [23, 75]. The findings from this RCT will determine if matched BCS dyads who receive 10 weeks of virtually delivered QEP support have higher levels of self-report and device-measured exercise, social support, and HRQOL compared to matched dyads without QEP-delivered exercise guidance.

Exercise interventions for cancer survivors led by a QEP, and using social support through matched dyads to facilitate exercise, have been separately documented in the literature [76–79]. To our knowledge this will be the first study to assess the combined effect of these two facilitators on exercise. Another unique component of this intervention is that the QEP support provided to the intervention group will be delivered virtually. There are numerous anticipated benefits to this intervention delivery method. First, the virtual delivery of exercise support is thought to meet the needs of diverse populations of cancer survivors, including those who are considered hard to reach (such as those who live in rural/remote areas and young adult cancer survivors) [25, 80]. For example, survivors living in rural areas often do not have access to a QEP trained in cancer rehabilitation and do not have a large community of cancer survivors to get support from and share experiences with [25, 81, 82]. Furthermore, young adult cancer survivors report difficulty accessing exercise services due to the timing and location of available exercise services which are not ideal due to their work and family commitments [25, 83]. Providing virtual delivery of exercise programming will make exercise services more available for many BCS as they will be able to access the intervention from anywhere, at more convenient times. However, since there will be cancer survivors who will not be able to access the internet, future work must consider the issue of technology equity and develop strategies to ensure equitable exercise-related services for this subgroup of survivors.

Virtual delivery of exercise support also improves the ability to match BCS dyads. Not having to limit matched dyads to those who live in the same geographical area is likely to lead to more well-matched dyads (i.e., matches will include those who have similar personal and cancer-related characteristics). This is important as survivors report needing exercise programs to include those of similar age and fitness level to motivate participation [25]. Furthermore, from a behavioral change perspective, support from similar others has been found to correlate with higher levels of exercise intention and behavior in cancer survivors [84]. Finally, delivering interventions virtually is vital in order to allow exercise services to continue safely during a global pandemic [85]. Currently, few virtually-delivered exercise interventions for cancer survivors are described in the literature [76, 86–88]. While the COVID-19 pandemic has been the impetus for clinicians to quickly change the way they practice, many components of these new healthcare delivery formats will likely continue post-pandemic. Therefore, it is important to evaluate these forms of virtual interventions.

This study will also examine the cost-effectiveness of the virtual QEP intervention for BCS dyads compared to traditional in-person QEP services, as well as a partner-based intervention without QEP support. Not only will these results have immediate implications for existing dyadic exercise support programs (such as ActiveMatch [activematch.ca] and 2Unstoppable [2unstoppable.org]), but together, the findings will help to determine the most cost-effective way to deliver exercise services to cancer survivors. This information is important to convey to healthcare policy makers as QEPs, clinicians, and researchers strive to make exercise a standard component of cancer care, in a healthcare system with limited funds and space [89].

## Limitations

Potential limitations should be taken into account when considering the prospective impact of this study. Specifically, all participants in this study will be English-speaking self-identified female BCS living in Canada. This limits the generalizability of the findings to survivors of other forms of cancer, male and intersex survivors, and non-Anglophones. This lack of generalizability is important to consider as exercise preferences are known to differ based on cancer type and sex. Also, the nature of distance-based interventions excludes those who do not have access to reliable internet and/or an internet-connected device, which may limit the usability of these findings for those individuals.

### Supplementary Information

**Additional file 1**. Outcome: Social support survey.

**Additional file 2**. Outcome: Use of health care services questionnaire.

**Additional file 3**. Outcome: Exercise peer-match quality quesitonnaire.

## Data Availability

Not applicable.

## Appendix: ***MatchQEP*** weekly discussion topics

**Table:**

Week	Topic	Actions/discussion points
1	Getting started	Exercise benefits Exercise guidelines Weekly exercise plan/schedule/introduce tracking exercise Introduce the RPE scale
2	Goal setting	The “5 why’s” exercise Goal setting and action planning F.I.T.T/A.I.M
3	Barriers	Barriers and obstacles Solutions If–then exercise
4	Social support	Connection Assess communication (what works/what doesn’t work) Types of support and strategies
5	Self-talk	All or nothing mindset Self-compassion statements Self-talk and mindfulness
6	Importance of recovery and rest	Yoga and stretching Sleep guidelines How to recognize stress and struggling
7	Habit forming	Habit stacking Increasing/decreasing friction Triggers and cues
8	How to progress	Exercise progression strategies Exercise principles (adaptation, progressive overlad, etc.) How to increase daily activity (NEAT) Maintenance is progress
9	Maintaining motivation	Reflect on motivation Future goal planning Short, medium, long term goals (post QEP sessions)
10	Relapse plan	Comeback plan

*RPE* rated of perceived exertion, *F.I.T.T* frequency, intensity, time, type, *A.I.M* accoutability, important, and measureable, *NEAT* non-exercise activity thermogenesis, *QEP* qualified exercise professional.

## Abbreviations

QEP: Qualified exercise professional
BCS: Breast cancer survivor
HRQOL: Health related quality of lfie
RCT: Randomized controlled trial
MVPA: Moderate-vigorous physical activity
APIM: Actor partner interdependence models
P1: Peer 1
P2: Peer 2
*ß*: Beta
*N*: Number
SSS: Social support survey
SF-12: Short form-12
EQ-5D-3L: EuroQOL-5 dimension-3 level
WAI-SR: Work alliance inventory-short form revised
