# Supplementary material for: Connecting breast cancer survivors for exercise: protocol for a two-arm randomized controlled trial
Source: BMC Sports Sci Med Rehabil. 2021 Oct 14;13:128. doi: 10.1186/s13102-021-00341-w (PMC8515152; doi:10.1186/s13102-021-00341-w)
Supplement: Supplementary file 1 — Additional file 1. Outcome: Social support survey. [file 13102_2021_341_MOESM1_ESM.docx]

**Additional File 1: SOCIAL SUPPORT SURVEY**

**OVERALL SOCIAL SUPPORT**

Currently, how satisfied are you with the **amount** of social support you receive overall?

| 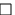 | 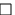 | 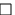 | 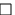 | 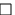 |  |
| --- | --- | --- | --- | --- | --- |
| Very dissatisfied | Dissatisfied | Neither satisfied nor dissatisfied | Satisfied | Very satisfied |  |

Currently, how satisfied are you with the **quality**of social support you receive overall?

| 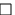 | 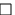 | 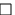 | 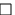 | 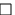 |  |
| --- | --- | --- | --- | --- | --- |
| Very dissatisfied | Dissatisfied | Neither satisfied nor dissatisfied | Satisfied | Very satisfied |  |

The following questions focus on individuals in your environment who provide you with help and/or support. Read the definition of the type of support being considered and answer all the questions as best you can.

| **Listening support:**People who listen to you without giving advice or being judgmental |
| --- |
| 1. How many individuals provide you with listening support?            0-1                       2-3                          4-5                     6-7                    8 or more         🞎                         🞎                            🞎                      🞎                       🞎⬝ |
| 1. In general, how satisfied are you with the overall quality of listening support you receive?     Very dissatisfied                                                                                     Very satisfied  🞎                         🞎                            🞎                      🞎                       🞎⬝ |

| **Task challenge:** People who challenge your way of thinking about your work or activity in order to stretch you, motivate you, and lead you to greater creativity, excitement, and involvement in your work or activity. |
| --- |
| 1. How many individuals provide you with task challenge support?            0-1                       2-3                          4-5                     6-7                    8 or more         🞎                         🞎                            🞎                      🞎                       🞎⬝ |
| 1. In general, how satisfied are you with the overall quality of task challenge support you receive?     Very dissatisfied                                                                               Very satisfied  🞎                         🞎                            🞎                      🞎                       🞎⬝ |

| **Emotional support:** People who comfort you and indicate to you that they are on your side and care for you. |
| --- |
| 1. How many individuals provide you with emotional support?            0-1                       2-3                          4-5                     6-7                    8 or more         🞎                         🞎                            🞎                      🞎                       🞎⬝ |
| 1. In general, how satisfied are you with the overall quality of emotional support you receive?            Very dissatisfied                                                                                     Very satisfied  🞎                         🞎                            🞎                      🞎                       🞎⬝ |

| **Esteem Support:** People providing encouragement that helps you in your work or activities. |
| --- |
| 1. How many individuals provide you with esteem support?            0-1                       2-3                          4-5                     6-7                    8 or more         🞎                         🞎                            🞎                      🞎                       🞎⬝ |
| 1. In general, how satisfied are you with the overall quality of esteem support you receive?     Very dissatisfied                                                                                     Very satisfied  🞎                         🞎                            🞎                      🞎                       🞎⬝ |

| **Reality confirmation:** People who are similar to you -- see things the way you do -- who help you confirm your perceptions and perspectives of the world and help you keep things in focus. |
| --- |
| 1. How many individuals provide you with reality confirmation support?            0-1                       2-3                          4-5                     6-7                    8 or more         🞎                         🞎                            🞎                      🞎                       🞎⬝ |
| 1. In general, how satisfied are you with the overall quality of reality confirmation support you receive?            Very dissatisfied                                                                                     Very satisfied  🞎                         🞎                            🞎                      🞎                       🞎⬝ |

| **Tangible assistance:** People who provide you with either financial assistance, products, and/or gifts. |
| --- |
| 1. How many individuals provide you with tangible assistance support?            0-1                       2-3                          4-5                     6-7                    8 or more         🞎                         🞎                            🞎                      🞎                       🞎⬝ |
| 1. In general, how satisfied are you with the overall quality of tangible assistance support you receive?     Very dissatisfied                                                                                     Very satisfied  🞎                         🞎                            🞎                      🞎                       🞎⬝ |

| **Understanding breast cancer support:** People who support you by letting you know that they understand what it is like to have breast cancer or have gone through breast cancer. |
| --- |
| 1. How many individuals provide you with understanding breast cancer support?            0-1                       2-3                          4-5                     6-7                    8 or more         🞎                         🞎                            🞎                      🞎                       🞎⬝ |
| 1. In general, how satisfied are you with the overall quality of understanding breast cancer support you receive?     Very dissatisfied                                                                                     Very satisfied  🞎                         🞎                            🞎                      🞎                       🞎⬝ |

**EXERCISE-RELATED SOCIAL SUPPORT**

Currently, how satisfied are you with the **amount** of support you receive **related to exercise**?

| 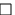 | 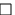 | 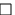 | 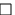 | 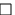 |  |
| --- | --- | --- | --- | --- | --- |
| Very dissatisfied | Dissatisfied | Neither satisfied nor dissatisfied | Satisfied | Very satisfied |  |

Currently, how satisfied are you with the **quality**of support you receive **related to exercise**?

| 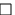 | 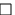 | 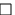 | 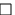 | 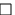 |  |
| --- | --- | --- | --- | --- | --- |
| Very dissatisfied | Dissatisfied | Neither satisfied nor dissatisfied | Satisfied | Very satisfied |  |

The following questions focus on individuals in your environment who provide you with help and/or support **related to exercise**. Read the definition of the type of support being considered and answer all the questions as best you can.

| **Listening support:**People who listen to you regarding **exercise**without giving advice or being judgmental |
| --- |
| 1. How many individuals provide you with listening support **regarding exercise**?            0-1                       2-3                          4-5                     6-7                    8 or more         🞎                         🞎                            🞎                      🞎                       🞎⬝ |
| 1. In general, how satisfied are you with the overall quality of listening support you receive **regarding exercise**??     Very dissatisfied                                                                                     Very satisfied  🞎                         🞎                            🞎                      🞎                       🞎⬝ |

| **Task challenge:** People who challenge your way of thinking about **exercise** in order to stretch you, motivate you, and lead you to greater creativity, excitement, and involvement in **exercise**. |
| --- |
| 1. How many individuals provide you with task challenge support?            0-1                       2-3                          4-5                     6-7                    8 or more         🞎                         🞎                            🞎                      🞎                       🞎⬝ |
| 1. In general, how satisfied are you with the overall quality of task challenge support you receive?     Very dissatisfied                                                                               Very satisfied  🞎                         🞎                            🞎                      🞎                       🞎⬝ |

| **Emotional support:** People providing empathy or care when you discuss the difficulties of exercise. |
| --- |
| 1. How many individuals provide you with emotional support around exercise?            0-1                       2-3                          4-5                     6-7                    8 or more         🞎                         🞎                            🞎                      🞎                       🞎⬝ |
| 1. In general, how satisfied are you with the overall quality of emotional support you receive around exercise?            Very dissatisfied                                                                                     Very satisfied  🞎                         🞎                            🞎                      🞎                       🞎⬝ |

| **Esteem Support:** People providing encouragement that helps you to exercise |
| --- |
| 1. How many individuals provide you with esteem support regarding exercise?            0-1                       2-3                          4-5                     6-7                    8 or more         🞎                         🞎                            🞎                      🞎                       🞎⬝ |
| 1. In general, how satisfied are you with the overall quality of esteem support you receive regarding exercise?     Very dissatisfied                                                                                     Very satisfied  🞎                         🞎                            🞎                      🞎                       🞎⬝ |

| **Reality confirmation:** People who are similar to you -- see things regarding exercise the way you do -- who help you confirm your perceptions and perspectives of exercise and help you keep things in focus. |
| --- |
| 1. How many individuals provide you with reality confirmation support?            0-1                       2-3                          4-5                     6-7                    8 or more         🞎                         🞎                            🞎                      🞎                       🞎⬝ |
| 1. In general, how satisfied are you with the overall quality of reality confirmation support you receive?            Very dissatisfied                                                                                     Very satisfied  🞎                         🞎                            🞎                      🞎                       🞎⬝ |

| **Tangible assistance:** People giving you materials or products that help you to exercise. |
| --- |
| 1. How many individuals provide you with tangible assistance support regarding exercise?            0-1                       2-3                          4-5                     6-7                    8 or more         🞎                         🞎                            🞎                      🞎                       🞎⬝ |
| 1. In general, how satisfied are you with the overall quality of tangible assistance support you receive regarding exercise?     Very dissatisfied                                                                                     Very satisfied  🞎                         🞎                            🞎                      🞎                       🞎⬝ |

| **Informational Support:** People providing information on the benefits of exercise |
| --- |
| 1. How many individuals provide you with informational support regarding exercise?            0-1                       2-3                          4-5                     6-7                    8 or more         🞎                         🞎                            🞎                      🞎                       🞎⬝ |
| 1. In general, how satisfied are you with the overall quality of informational support you receive regarding exercise?     Very dissatisfied                                                                                     Very satisfied  🞎                         🞎                            🞎                      🞎                       🞎⬝ |

***For post-intervention, post-tapering, and follow-up assessment time points only:***

How much of each type of exercise-related support have you received from your **study** **exercise partner**since your last assessment time point?

| **Tangible assistance**    (e.g., gave you materials or products that helped you to exercise) | None at all  1 | 2 | 3 | 4 | 5 | 6 | A lot  7 |
| --- | --- | --- | --- | --- | --- | --- | --- |
| **Emotional support**    (e.g., provided empathy or care when you discussed the difficulties of exercise) | None at all  1 | 2 | 3 | 4 | 5 | 6 | A lot  7 |
| **Informational support**    (e.g., provided information on the benefits of exercise) | None at all  1 | 2 | 3 | 4 | 5 | 6 | A lot  7 |
| **Esteem support**    (e.g., encouraged you to exercise) | None at all  1 | 2 | 3 | 4 | 5 | 6 | A lot  7 |
