# Supplementary material for: Connecting breast cancer survivors for exercise: protocol for a two-arm randomized controlled trial
Source: BMC Sports Sci Med Rehabil. 2021 Oct 14;13:128. doi: 10.1186/s13102-021-00341-w (PMC8515152; doi:10.1186/s13102-021-00341-w)
Supplement: Supplementary file 2 — Additional file 2. Outcome: Use of health care services questionnaire. [file 13102_2021_341_MOESM2_ESM.docx]

**Additional File 2: USE OF HEALTH CARE SERVICES** **QUESTIONNAIRE**

Please list all of the times you have used health care services **that are outside of your usual cancer treatment**since your last assessment.

**Health Care Facility / Doctor Visit:**

| **Health care category:** | **Total number of visits *since last assessment*:** |
| --- | --- |
| Walk in clinic: |  |
| Urgent care service: |  |
| Emergency room: |  |
| Family doctor visit: |  |
| Specialist (oncologist / surgeon) visit: |  |

**Procedures:**

| **Type of procedure:** | **Total number of procedures *since last assessment:*** |
| --- | --- |
| Blood test: |  |
| X-ray: |  |
| MRI: |  |
| CT Scan: |  |
| Ultrasound: |  |
| Minor surgery:  *Please specify type: ________*  *Please specify type: ________*  *Please specify type: ________* |  |
| Other:  *Please specify: _________*  *Please specify: _________*  *Please specify: _________* |  |

**Support Services:**

Please describe any support services you have received since your last assessment.

| **Type of service:** | **Number of sessions *since last assessment*:** | **Payment method (please select):** |
| --- | --- | --- |
| Social Worker: |  | Provincial Health Insurance; Private Insurance; Out of Pocket |
| Personal Support Worker: |  | Provincial Health Insurance; Private Insurance; Out of Pocket |
| Registered Nurse: |  | Provincial Health Insurance; Private Insurance; Out of Pocket |
| Occupational Therapist: |  | Provincial Health Insurance; Private Insurance; Out of Pocket |
| Physiotherapist: |  | Provincial Health Insurance; Private Insurance; Out of Pocket |
| Registered Kinesiologist: |  | Provincial Health Insurance; Private Insurance; Out of Pocket |
| Psychologist: |  | Provincial Health Insurance; Private Insurance; Out of Pocket |

**Loss of Work:**

Please specify how your health has impacted your ability to work, and your family’s ability to work, ***since your last assessment***.

| ***Yourself*:** | | | |
| --- | --- | --- | --- |
| Current Occupation: | | | |
| Employment Status: | - Full time employed - Part time employed - Retired - Unemployed - Student - Disability - Sick leave | | |
| Loss of work: Have you missed work since your last assessment (10 weeks ago)? Yes ☐ , No ☐.  If yes, how many days?:  How many of these days were cancer related?: | | | |
| ***Family Member:*** | | | |
| Loss of work: Has any family member missed any work to look after you or take you to appointments since your last assessment (10 weeks ago)? Yes ☐, No ☐    If yes, indicate total hours and occupation for each different family member that helped you: | | | |
| Family Member: | | Total Hours | Occupation |
| 1 | |  |  |
| 2 | |  |  |
| 3 | |  |  |
| 4 | |  |  |
