# Supplementary material for: Connecting breast cancer survivors for exercise: protocol for a two-arm randomized controlled trial
Source: BMC Sports Sci Med Rehabil. 2021 Oct 14;13:128. doi: 10.1186/s13102-021-00341-w (PMC8515152; doi:10.1186/s13102-021-00341-w)
Supplement: Supplementary file 3 — Additional file 3. Outcome: Exercise peer-match quality quesitonnaire. [file 13102_2021_341_MOESM3_ESM.docx]

**Additional File 3: EXERCISE PEER-MATCH QUALITY QUESTIONNAIRE**

**YOUR EXERCISE PARTNER**

How satisfied are you with the **quality of your exercise partner match**to support exercise in this project?

| 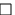 | 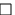 | 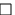 | 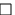 | 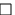 |  |
| --- | --- | --- | --- | --- | --- |
| Very dissatisfied | Dissatisfied | Neither satisfied nor dissatisfied | Satisfied | Very satisfied |  |

To what extent is/was your assigned exercise partner a good match for you?

| 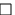 | 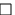 | | 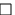 | | 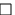 | | 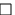 | | |
| --- | --- | --- | --- | --- | --- | --- | --- | --- | --- |
| Very good match | | Good match | | Neither | | Poor match | | Very poor match |  |

Please explain:

To what extent are/were you and your exercise partner similar?

| 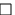 | 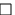 | 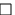 | 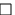 | 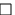 |
| --- | --- | --- | --- | --- |
| Extremely similar | Similar | Neither similar nor dissimilar | A little bit similar | Not at all similar |

Which characteristics, if any, do/did you and your exercise partner have in common? (Select all that apply)

- Age
- Stage of cancer at diagnosis
- Time since cancer diagnosis
- Treatments received
- Education
- Physical activity level pre-cancer
- Current physical activity level
- Other (please specify):

On average, how often did you communicate with your exercise partner each week, and for how long?

| Average number of times per week: | **______________________________________** |
| --- | --- |
| Average duration of each contact (minutes): | **______________________________________** |

How have you communicated with your partner? (Select all that apply)

- Email
- Text message
- Instant messaging (e.g., Facebook messenger)
- Phone call
- Video call
- In person
- Other:

What, if anything, made it difficult to communicate with or relate to your exercise partner?
